# Diagnostic stewardship for *Clostridioides difficile* testing in an acute care hospital: A quality improvement intervention

**DOI:** 10.1017/ash.2023.141

**Published:** 2023-04-05

**Authors:** Madeline L. Berg, Ashley M. Ayres, David R. Weber, Melissa McCullough, Victoria D. Crall, Casey L. Lewis, Abby L. Valek, Lizabeth A. Vincent, Joseph Penzelik, Crystal A. Sasinoski, Amanda L. Cheng, Claire F. Bradford, Elizabeth O. Bell, Kimberly M. Edwards, Isabella A. Castronova, Mya B. Brady, Julie Slaughter, Louise-Marie Oleksiuk, Graham M. Snyder

**Affiliations:** 1 Department of Infection Prevention and Control, UPMC Presbyterian/Shadyside, Pittsburgh, Pennsylvania; 2 Division of Laboratory Medicine, Department of Pathology, University of Pittsburgh Medical Center, Pittsburgh, Pennsylvania; 3 Department of Anesthesiology and Perioperative Medicine, UPMC Shadyside, Pittsburgh, Pennsylvania; 4 Department of Pharmacy, UPMC Presbyterian/Shadyside, Pittsburgh, Pennsylvania; 5 Division of Infectious Diseases, Department of Medicine, University of Pittsburgh School of Medicine, Pittsburgh, Pennsylvania

## Abstract

**Objective:**

To evaluate the impact of a diagnostic stewardship intervention on *Clostridioides difficile* healthcare-associated infections (HAI).

**Design:**

Quality improvement study.

**Setting:**

Two urban acute care hospitals.

**Interventions:**

All inpatient stool testing for *C. difficile* required review and approval prior to specimen processing in the laboratory. An infection preventionist reviewed all orders daily through chart review and conversations with nursing; orders meeting clinical criteria for testing were approved, orders not meeting clinical criteria were discussed with the ordering provider. The proportion of completed tests meeting clinical criteria for testing and the primary outcome of *C. difficile* HAI were compared before and after the intervention.

**Results:**

The frequency of completed *C. difficile* orders not meeting criteria was lower [146 (7.5%) of 1,958] in the intervention period (January 10, 2022–October 14, 2022) than in the sampled 3-month preintervention period [26 (21.0%) of 124; P < .001]. *C. difficile* HAI rates were 8.80 per 10,000 patient days prior to the intervention (March 1, 2021–January 9, 2022) and 7.69 per 10,000 patient days during the intervention period (incidence rate ratio, 0.87; 95% confidence interval, 0.73–1.05; P = .13).

**Conclusions:**

A stringent order-approval process reduced clinically nonindicated testing for *C. difficile* but did not significantly decrease HAIs.

*Clostridioides difficile* causes nearly 500,000 infections in the Unites States annually, and colonization without infection is prevalent among hospitalized patients, occurring in 4%–15% of individuals.^1,2^ *C. difficile* testing using molecular methods poorly differentiates between colonization and active infection, and clinical correlation is necessary to determine treatment indication.^1,3^

*C. difficile* healthcare-associated infections (HAIs) are reported to the CDC National Healthcare Safety Network (NHSN) for patients who test positive for *C. difficile* on hospital day 3 or later, regardless of the clinical assessment of infection.^4^ As a result, testing for *C. difficile* on patients who are clinically unlikely to have colitis may result in reporting of HAIs among patients who are asymptomatic carriers of *C. difficile*. Furthermore, clinically nonindicated treatment of *C. difficile* colonization may affect the diversity of the patient’s intestinal microbiome, increase subsequent risk for developing a *C. difficile* infection, increase the development of multidrug resistant organisms through inappropriate use of antibiotics, and increase length of stay and healthcare costs.^1^

Facility-level opportunities to reduce clinically nonindicated testing for *C. difficile* may consider passive decision support (eg, ordering criteria displayed at the time of order) to encourage clinically indicated testing, or administrative and laboratory controls (eg, systematically cancelling orders that are not indicated). Some studies showed that alerts successfully decreased clinically nonindicated orders.^5^ However, various studies have shown that administrative or laboratory test restriction for *C. difficile*, rather than passive decision support more effectively improve diagnostic stewardship of *C. difficile.* ^1,6–8^ Passive decision support via alerts can result in “alert fatigue,” in which providers no longer engage with the alert due to overfiring.^5^

Root-cause analyses performed prior to the intervention identified diagnostic stewardship as an opportunity for hospitals A and B. Unadjusted baseline *C. difficile* HAI rates for hospital A were 8.83 and 8.36 per 10,000 patient days in 2020 and 2021 and for hospital B these rates were 7.03 and 9.34 per 10,000 patient days in 2020 and 2021. Standardized infection ratios for *C. difficile* were 0.997 and 0.805 in 2020 and 2021 for hospital A and 0.844 and 0.941 in 2020 and 2021 for hospital B.^9^ In this quality improvement intervention, we aimed to decrease *C. difficile* HAIs through reduction of clinically nonindicated testing by infection prevention review and approval of all *C. difficile* orders placed in the hospital.

## Methods

### Study setting

This quality improvement intervention took place at 2 hospitals: UPMC Presbyterian (hospital A) and UPMC Shadyside (hospital B), located in Western Pennsylvania. Hospital A is a 695-bed, level 1 regional resource trauma center that specializes in solid-organ transplants; hospital B is a 520-bed, tertiary-care hospital specializing in oncology care. This study was granted approval as a quality improvement project by the UPMC Quality Improvement Review Committee (project no. 3808). The study population included inpatients at hospitals A and B who had *C. difficile* testing ordered between January 10, 2022, and October 14, 2022.

Prior to and throughout this intervention, *C. difficile* testing was recommended only after 2 criteria were met: (1) at least 3 loose stools in a 24-hour period not explained by laxatives, enteral feeding, enemas, or bowel preparation, and (2) at least 1 clinical indication of infection was present including antibiotic exposure within the prior 60 days (ie, body temperature >38°C, abdominal tenderness, cramping or distention, peripheral blood total white blood cell count >10,000 cells/µL, recent chemotherapy or current immunosuppression, or history of *C. difficile* infection). Since 2018, these criteria have been included in education, facility guidelines, and as a decision support tool in the electronic health record. *C. difficile* testing is only performed on stool that conforms to the shape of the sample container. *C. difficile* testing was performed using a 2-step testing algorithm.^10^ Enzyme immunoassay is performed to detect glutamate dehydrogenase and the toxin produced by *C. difficile* (Techlab, Blacksburg, VA) and a polymerase chain reaction test is performed to detect toxin gene production (Cepheid, Sunnyvale, CA) for discordant results.

### Study intervention

The intervention added the requirement for review by an infection preventionist for inpatient *C. difficile* orders prior to laboratory processing. After providers placed *C. difficile* orders and the sample was submitted to the clinical laboratory, specimen processing was held until the order was approved by an infection preventionist. All existing orders were reviewed by infection preventionists from 1:00–3:00 p.m. daily; orders placed after 3:00 p.m. were reviewed the next day. Infection preventionists performed chart reviews and reviewed the patient’s clinical status with the bedside nurse, as necessary, to determine whether the order met the existing ordering criteria. Orders meeting testing criteria were approved. If ordering criteria were not met, the infection preventionist contacted the ordering provider to discuss the order indication and provide education on diagnostic stewardship of *C. difficile*. If the provider believed after education that the order was still indicated, or the infection preventionist could not reach the ordering provider, the order was approved. Order status (“approved” or “not approved”) for held specimens were submitted to the laboratory at 3:00 p.m. daily and were processed the following morning. Specimens not approved for testing were discarded.

Inclusion criteria for the intervention included all inpatients being tested for *C. difficile*. The emergency departments and outpatient locations were excluded from the intervention and analysis.

Contact precautions were applied at the time of the order, and providers were encouraged to consider empiric therapy for *C. difficile* pending the test result.

This quality improvement intervention began on the oncology units at hospital B where an opportunity for diagnostic stewardship was identified through event reviews. A pilot project was initiated on hospital B oncology units from September 12, 2021, through October 1, 2021, and was reimplemented on October 22, 2021 and continued subsequently. After its success, the decision was made to expand the intervention to all inpatient units at hospitals A and B on January 10, 2022.

### Outcomes and data sources

The outcomes of the investigation were the proportion of completed tests meeting testing criteria and the primary outcome of *C. difficile* healthcare-associated infections, which are any positive *C. difficile* sample collected on or after hospital day 3.^4^ Additional outcomes included potential adverse events of canceled testing, characterized by testing positive for *C. difficile* after having an order canceled with the intervention, and the exploratory outcome of antimicrobial use targeting *C. difficile* infection. For antimicrobial use, we hypothesized that the rate of antimicrobial use may not change because we did not expect to decrease true infections and because providers are educated not to treat *C. difficile* colonization cases.

The intervention period for this study was January 10, 2022, through October 14, 2022. Because this study was a quality improvement project that started with the pilot project in October, baseline (preintervention) data on *C. difficile* orders were collected from October 1, 2021, through January 9, 2022. The baseline period for HAI and antimicrobial usage outcomes was March 1, 2021–January 9, 2022. *C. difficile* HAIs and antimicrobial usage were reported monthly. The additional outcomes representing process measures were analyzed using weekly intervals consistent with how these data were used for quality improvement efforts. Intervention weeks are oriented according to intervention date: weeks −15 through −1 for the preintervention period and weeks 1 through 40 for the intervention period. Weeks −15 and 40 were partial weeks because the overall quality improvement analysis included events occurring October 2021 through October 14, 2022. Data from units participating in the pilot project were included with hospital-wide preintervention data.

During the preintervention period, and to assess order appropriateness in the preintervention period only, a random sample comprising 15% of completed inpatient *C. difficile* orders (resulting positive or negative) was reviewed for order appropriateness, and was assessed using the described *C. difficile* ordering criteria. By the nature of the intervention, all orders during the intervention period were evaluated for appropriateness. To identify adverse outcomes attributed to delayed diagnoses of *C. difficile*, records were reviewed for *C. difficile* infection diagnoses for 30 days following the canceled order among patients with an order that was not approved for testing. Facility-wide prescription of antimicrobials targeting *C. difficile* including vancomycin oral formulation and fidaxomicin were quantified for the preintervention and intervention periods, calculated as monthly days of therapy per 1,000 patient days. Data were collected from the electronic health record and existing infection prevention and antimicrobial stewardship quality improvement databases and were stored and analyzed using Microsoft Excel (Microsoft, Redmond, WA). For orders that were canceled with the intervention, reasons testing was no longer indicated as well as subsequent *C. difficile* testing or diagnoses within 30 days were recorded.

### Statistical methods

To demonstrate the ability of the intervention to decrease nonindicated orders, we compared the proportion of completed orders not meeting ordering criteria before and after intervention. We compared the rate of *C. difficile* HAI (events per 10,000 patient days) in the preintervention and intervention periods by calculating an incidence rate ratio. Because this was a quality improvement intervention with prespecified trial periods, we did not perform sample size or power calculations prior to the intervention. *P* values were calculated using median-unbiased estimation. We describe potential adverse events attributable to tests canceled by the intervention. Because *C. difficile*–targeted antimicrobial therapy was examined in an exploratory analysis, we have reported days of therapy for vancomycin and fidaxomicin in the preintervention and intervention periods. Statistical calculations were performed using Stata version 12.1 software (StataCorp, College Station, TX).

## Results

During the intervention period (January 1, 2022–October 14, 2022), infection preventionists reviewed 3,395 *C. difficile* orders, of which 429 (12.6%) did not meet clinical criteria. Of the 429 orders that did not meet the criteria, 217 (50.6%) were canceled by the intervention (Table 1). For the remaining 212 orders, 96 were approved because an infection preventionist could not reach the ordering provider and 116 were approved because the ordering provider disagreed with the infection preventionist. Also, 20 HAIs were reported on these 212 orders that the infection preventionist assessed did not meet criteria. Order outcomes of approved orders are reported in Table 2. The reasons orders were cancelled by the intervention are described in Table 3.

**Table 1.** *Clostridioides difficile* Order Review Outcomes During the Intervention Period, by Intervention Hospital

Review Outcome	Hospital A (N = 2,053)		Hospital B (N = 1,342)		Total (N = 3,395)	
	No.	(%)	No.	(%)	No.	(%)
Order met criteria and approved	1,447	(70.5)	1,080	(80.5)	2,527	(74.4)
Order did not meet criteria	256	(12.5)	173	(12.9)	429	(12.6)
Approved for processing	*124*	*(48.4)*	*88*	*(50.9)*	*212*	*(49.4)*
Not approved and canceled	*132*	*(51.6)*	*85*	*(49.1)*	*217*	*(50.6)*
Duplicate order^a^	319	(15.5)	64	(4.8)	383	(11.3)
Provider already canceled	12	(0.6)	23	(1.7)	35	(1.0)
Laboratory processed without approval^b^	19	(0.9)	2	(0.1)	21	(0.6)

a Duplicate orders are orders placed again after infection prevention approved an order in the day or 2 prior days.

b 21 samples were accidentally processed without waiting for infection prevention approval.

**Table 2.** *Clostridioides difficile* Inpatient Order Outcomes of Approved Orders During the Intervention Period, by Intervention Hospital

Order Outcome	Hospital A^a^ (N = 1,571)		Hospital B (N = 1,168)		Total (N = 2,739)	
	No.	(%)	No.	(%)	No.	(%)
**Order met criteria**	1,447	(92.1)	1,080	(92.5)	2,527	(92.3)
Negative	875	(60.5)	688	(63.7)	1,563	(61.9)
Not collected	422	(29.2)	283	(26.2)	705	(27.9)
Positive, healthcare-associated infection	115	(7.9)	77	(7.1)	192	(7.6)
Positive, present on admission	25	(1.7)	32	(3.0)	57	(2.3)
Rejected by the laboratory	10	(0.7)	0	(0.0)	10	(0.4)
**Order did not meet criteria**	124	(7.9)	88	(7.5)	212	(7.7)
Negative	73	(58.9)	51	(58.0)	124	(58.5)
Not collected	41	(33.1)	24	(27.3)	65	(30.7)
Positive, healthcare-associated infection	8	(6.5)	12	(13.6)	20	(9.4)
Positive, present on admission	1	(0.8)	1	(1.1)	2	(0.9)
Rejected by the laboratory	1	(0.8)	0	(0.0)	1	(0.5)

a 3 other *C. difficile* HAIs were reported during this period for hospital A: 2 were from orders placed at an outside facility and 1 was on an order that the laboratory processed without infection prevention approval.

**Table 3.** Reasons *Clostridioides difficile* Orders Were Not Approved During the Intervention, by Intervention Hospital

Order Nonapproval Indication	Hospital A (N = 132)		Hospital B (N = 85)		Total (N = 217)	
	No.	(%)	No.	(%)	No.	(%)
Diarrhea resolved	36	(27.3)	28	(32.9)	64	(29.5)
Laxative exposure	28	(21.2)	20	(23.5)	48	(22.1)
Did not have at least 3 loose stools within 24 h	18	(13.6)	16	(18.8)	34	(15.7)
Previous negative test	22	(16.7)	9	(10.6)	31	(14.3)
Diarrhea related to enteral feeding	12	(9.1)	1	(1.2)	13	(6.0)
Previous positive test	9	(6.8)	4	(4.7)	13	(6.0)
Already being treated for *C. difficile*	3	(2.3)	2	(2.4)	5	(2.3)
Alternative diagnosis	2	(1.5)	2	(2.4)	4	(1.8)
Patient deceased at time of order	0	(0.0)	2	(2.4)	2	(0.9)
Diarrhea due to enema	2	(1.5)	0	(0.0)	2	(0.9)
Deceased at time of order	0	(0.0)	1	(1.2)	1	(0.5)

### Clinically nonindicated (completed) orders

**Fig. 1.**
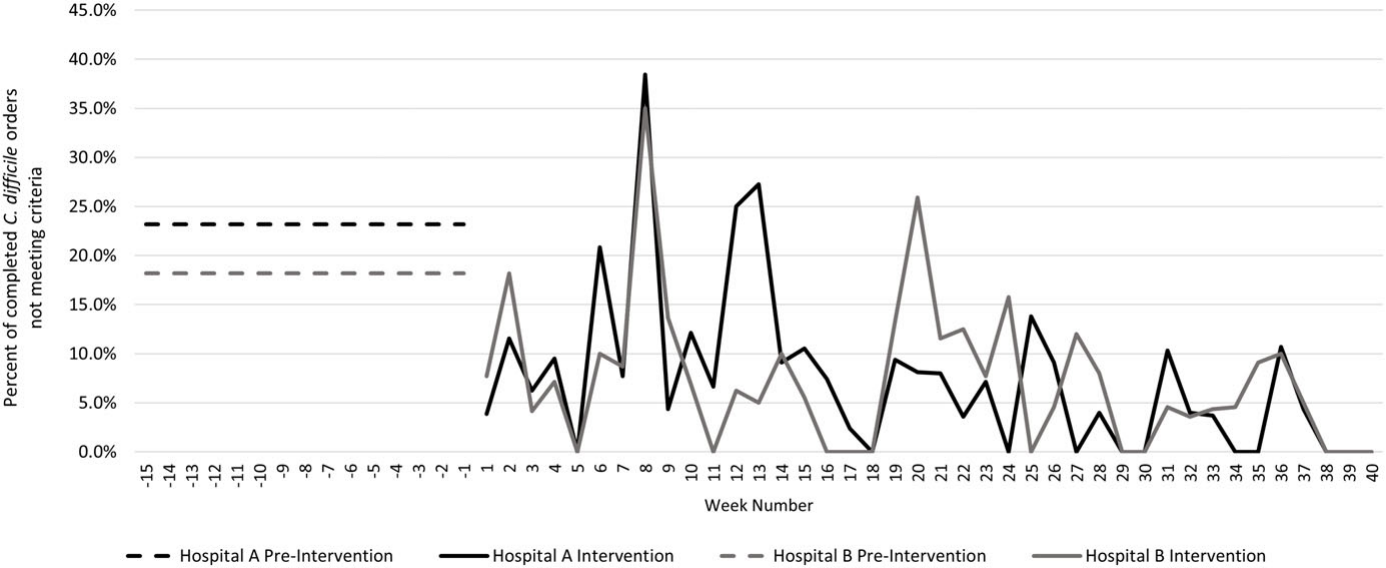
Proportion of completed *Clostridioides difficile* orders not meeting criteria for appropriateness. The figure excludes 19 completed orders that were processed without infection prevention approval.

During the preintervention period (October 1, 2021–January 9, 2022), 823 inpatient orders were placed and completed (resulting positive or negative): 458 (55.7%) at hospital A and 365 (44.3%) at hospital B. The 15% random sampling of preintervention orders comprised 124 orders; among them, 26 (21.0%) did not meet the ordering criteria. During the intervention period, 1,958 orders were completed, of which 146 did not meet ordering criteria (Supplementary Table S1). The frequency of completed *C. difficile* orders not meeting criteria was lower during the intervention period [146 (7.5%) of 1,958] than in the sampled preintervention period [26 (21.0%) of 124; *P* value for comparison, <.001] (Fig. 1).

#### Clostridioides difficile *HAI*

**Fig 2.**
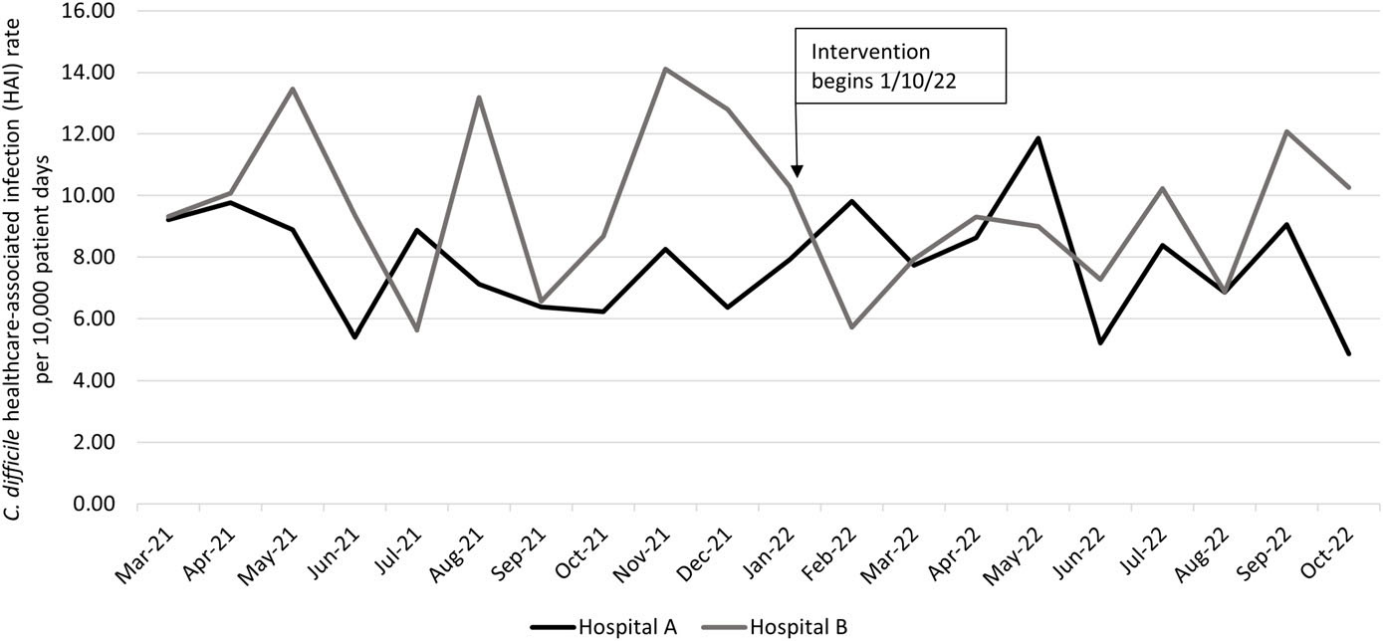
*Clostridioides difficile* healthcare-associated infections in the preintervention and intervention periods, monthly by hospital.

*Clostridioides difficile* HAI counts are reported in Supplementary Table S2. Figure 2 depicts the change in rate of *C. difficile* HAI per 10,000 patient days from the preintervention period to the intervention period. The incidence rate ratio of *C. difficile* HAI was 0.87 (95% confidence interval [CI], 0.73–1.05; *P* value, 0.13), with overall HAI rates of 8.80 per 10,000 patient days (279 HAI in 316,893 patient days) in the preintervention period and 7.69 per 10,000 patient days (214 HAI in 278,444 patient days) in the intervention period. At hospital A, the incidence rate ratio was 0.92 (95% CI, 0.72–1.17; *P =* .47); at hospital B, the incidence rate ratio was 0.83 (95% CI, 0.62–1.10; *P =* .18).

In total, 45 orders led to subsequent *C. difficile* tests completed after the initial order was canceled for not meeting ordering criteria in the intervention, 7 of which were positive (Table 4). Among them, 2 clinically represented colonization in which 1 patient was treated and 1 patient was not; 3 patients had resolution of diarrhea at the time of the first order and a recurrent episode of diarrhea prompting the reorder. The sixth patient had an order canceled due to laxative exposure and then was positive on retesting after diarrhea did not resolve despite holding laxatives. The seventh patient had diarrhea while on enteral feeding and tested positive after diarrhea continued despite a hold on enteral feeding. We did not observe any *C. difficile*–attributable intensive care unit admission, surgical intervention, or death among patients with an order canceled by the intervention. None of these patients were observed to meet *C. difficile* ordering criteria within a few days of order cancelation.

**Table 4.** Outcomes of Retesting Following Initial Intervention-Canceled *Clostridioides difficile* Order

Retesting Outcome	Hospital A (N = 132)		Hospital B (N = 85)		Total (N = 217)	
	No.	(%)	No.	(%)	No.	(%)
Not Retested	107	(81.1)	65	76.5)	172	(79.3)
Retested positive	4	(3.0)	3	(3.5)	7	(3.2)
Retested negative	21	(15.9)	17	(20.0)	38	(17.5)

#### Clostridioides difficile *antimicrobial days of therapy*

**Fig 3.**
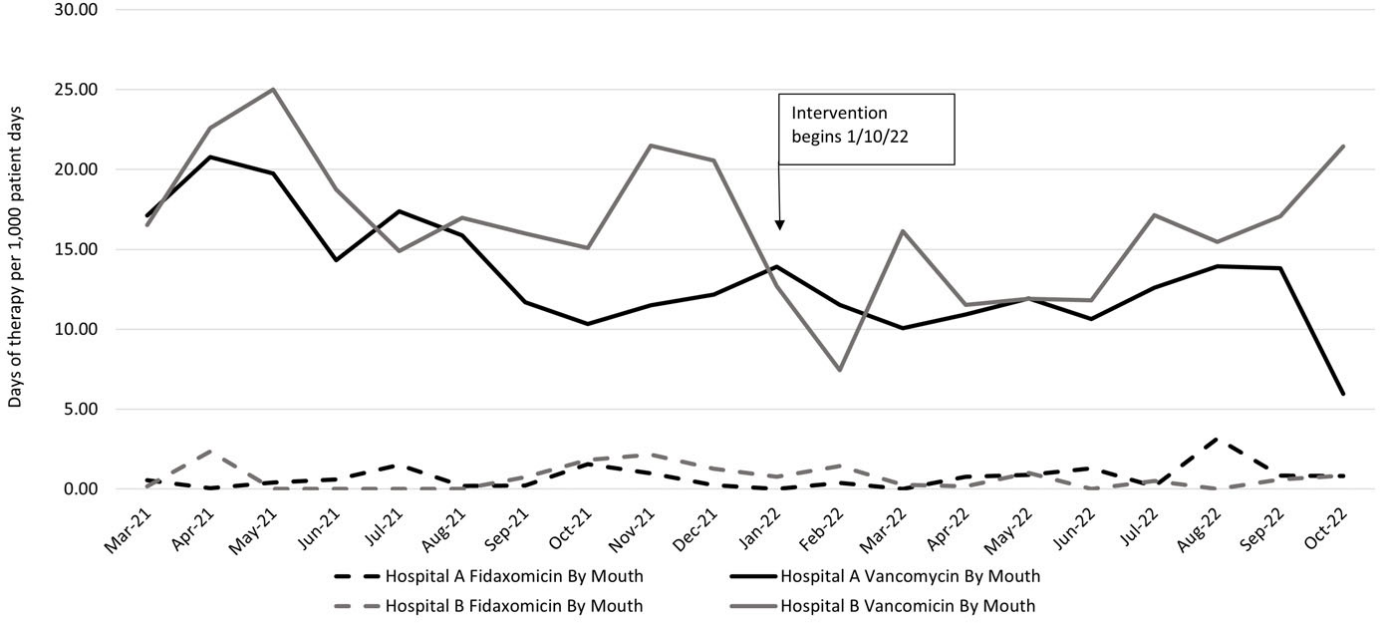
Vancomycin and fidaxomicin usage in the preintervention and intervention periods, monthly by hospital.

Oral vancomycin and fidaxomicin days of therapy per 1,000 patient days at hospitals A and B are described in Figure 3. Days of therapy for oral vancomycin usage decreased from 14.92 per 1,000 patient days to 11.55 per 1,000 patient days at hospital A, and oral vancomycin usage decreased from 18.74 per 1,000 patient days to 14.40 per 1,000 patient days at hospital B.

## Discussion

We implemented a diagnostic stewardship intervention in which every inpatient *C. difficile* order was reviewed by an infection preventionist for clinical appropriateness criteria. Orders not meeting criteria decreased, although they were not eliminated. *C. difficile* HAIs showed a nonsignificant 8%–17% decrease in incidence. The intervention did not result in any adverse events attributable to order cancelation. This intervention was expected to be a strong diagnostic stewardship intervention but resulted in only modest impacts on patient and health system–oriented outcomes.

This intervention successfully decreased clinically nonindicated orders; however, only half of the clinically nonindicated orders were canceled in the intervention. Barriers to a more effective intervention are instructive: infection preventionists approved some orders for processing due to not being able to reach the ordering provider, and in other cases the provider believed testing was still indicated despite not meeting evidence-based criteria for nontesting. Diagnostic stewardship interventions to improve *C. difficile* testing practices may require more tailored approaches including accountability, normalization to peer practice, changing outcome expectations, and culture change.^11,12^

After intervention implementation, HAIs decreased nonsignificantly at both hospitals A and B. Because data from the hospital B pilot intervention were included with the preintervention data, this difference may have been underestimated. If facilities are considering intervention, a higher cancellation rate of orders not meeting criteria may be needed to have success in HAI reduction. A smaller-than-expected decrease in HAIs in the intervention period may be attributable to delay in diagnosis, transmission, or systemic antibiotic use. Additionally, 20 orders that the infection preventionist was unable to cancel for not meeting criteria resulted positive as HAIs. Which also may have prevented a larger decrease in HAI rate from being observed during the intervention period. Days of therapy for oral vancomycin usage decreased at both hospitals A and B. We expected usage to stay the same because providers are taught to only treat for true infection and our intervention aimed to decrease HAIs that are reported on colonization cases. Antimicrobial stewardship initiatives in place during this period may have resulted in a decrease in rate.

Many patients who underwent retesting after initial order cancellation had a negative test or a positive test that may have been interpreted as colonization. This finding reinforces the utility of the clinical criteria for testing.^9^ We identified 2 instances of true delay in diagnosis of colitis, in patients whose *C. difficile* order was considered clinically nonindicated due to alternative causes of diarrhea (laxative and enteral feeding exposure); no adverse outcomes as a result of the delay were observed with these patients. Also, 58 other patients exposed to laxatives or enteral feeding who had orders canceled with the intervention did not test positive within 30 days of order cancellation, affirming the value of this clinical criterion to avoid testing.^9^ The most common reason orders did not meet criteria was that the patient had a resolution in diarrhea (30%). Because infection preventionists reviewed orders from 1:00 to 3:00 p.m. daily and not in real time, there was opportunity to notice a resolution in loose stools in some patients before orders were reviewed. Although a delay in diagnosis and treatment of *C. difficile* may result in a worse outcome, this finding points to a potential need for a more focused and objective observation of clinical findings (namely, stool frequency and consistency) before placing a *C. difficile* order.

Various studies have used similar models to improve diagnostic stewardship of *C. difficile*. Two studies of antimicrobial stewardship pharmacist approval of *C. difficile* orders showed a significant decrease in orders^7^ and a significant reduction in *C. difficile* HAIs after implementation.^13^ In 2 studies that utilized infection prevention approval of orders, decreases occurred in testing and *C. difficile* HAIs.^14,15^ Yet our study did not show a significant decrease in *C. difficile* HAIs. This could have been due to the unique patient populations of hospitals A and B, specializing in transplant and oncology patients. Furthermore, this intervention was not a part of a “bundle,” so the impact of the intervention may have been weak in isolation. Also, our study did not impose a true “hard stop” on the order; orders were still allowed to go through if providers disagreed with the infection preventionist or if providers did not answer calls about orders.

A large team of 12 infection preventionists reviewed orders, which allowed for implementation of this resource-intensive intervention; smaller facilities may not be able to adopt this strategy. We also noted variation among infection preventionists in the cancellation rate for orders that did not meet criteria (data not shown). If considering this intervention, performance evaluation and training of those doing the reviews may be necessary for success. Support from hospital leadership encouraging prescriber participation likely improved the impact of the intervention. The supplement provides more information on our quality improvement learnings.

Our intervention and analysis are subject to limitations. As a before-and-after study design, underlying temporal trends may not be accounted for. Our analysis may have underestimated the effect estimate at hospital B because the unit-restricted pilot was included in the preintervention data. A larger sample size may be necessary to observe a small effect of the intervention on *C. difficile* HAI. One effect of this intervention may have been to decrease clinically nonindicated orders from being placed in the first place, and although we did not measure this impact directly, a lower frequency of orders that are clinically nonindicated in the intervention period (12.6%) than in the preintervention period (18%–23%) suggests that this may be the case.

Despite not having the predicted impact on HAI reduction, this diagnostic stewardship project successfully decreased clinically nonindicated orders. In the future, more work is needed to create a less time-intensive or an automated process for preventing clinically nonindicated testing.

## Supplementary material

For supplementary material accompanying this paper visit http://doi.org/10.1017/ash.2023.141. click here to view supplementary material
