# Supplementary material for: Diagnostic stewardship for Clostridioides difficile testing in an acute care hospital: A quality improvement intervention
Source: Antimicrob Steward Healthc Epidemiol. 2023 Apr 5;3(1):e67. doi: 10.1017/ash.2023.141 (PMC10127245; doi:10.1017/ash.2023.141)
Supplement: Supplementary file 1 [file ashsup.zip › S2732494X23001419sup001.pdf]

Diagnostic stewardship for *Clostridioides difficile* testing in an acute care hospital: A quality improvement intervention

Supplemental Digital Content 1: Strengthening the Reporting of Observational Studies in Epidemiology (STROBE) and Standards for Quality Improvement Reporting Excellence (SQUIRE) 2.0 Guidelines

	Item No.	STROBE items	Location	SQUIRE items	Location
**Title and Abstract**					
	1	(a) Indicate the study's design with a commonly used term in the title or the abstract (b) Provide in the abstract an informative and balanced summary of what was done and what was found	a) Abstract - Design<br><br>b) Abstract – Methods, Results	Title: Indicate that the article concerns an initiative to improve healthcare.<br><br>Abstract: This is a summary of your work and is the most important section to attract a reader's attention. Please ensure you include a brief background to the problem, the method for your quality improvement project, the overall results and conclusion.	Title line and abstract section (brief background not included in abstract)
**Introduction**					
Background rationale	2	Explain the scientific background and rationale for the investigation being reported	Introduction – paragraph 2, 3, 4	Background information about the problem and up-to-date, research and knowledge from the literature.	Introduction – paragraph 2, 3, 4
Objectives	3	State specific objectives, including any prespecified hypotheses	Introduction – paragraph 4 – last sentence	Summarize your problem and the focus of your project.	Introduction – paragraph 4
**Methods**					
Study Design	4	Present key elements of study design early in the paper	Methods – Study Intervention, paragraph 1	Describe any reasons or assumptions that were used to develop the intervention(s) and reasons why you expected them to work.	Not described in the methods

Setting	5	Describe the setting, locations, and relevant dates, including periods of recruitment, exposure, follow-up, and data collection	Methods – Study setting, paragraph 1, - Outcomes and data sources, paragraph 2		
Participants	6	*(a) Cohort study* - Give the eligibility criteria, and the sources and methods of selection of participants. Describe methods of follow-up<br>*Case-control study* - Give the eligibility criteria, and the sources and methods of case ascertainment and control selection. Give the rationale for the choice of cases and controls<br>*Cross-sectional study* - Give the eligibility criteria, and the sources and methods of selection of participants<br><br>*(b) Cohort study* - For matched studies, give matching criteria and number of exposed and unexposed<br>*Case-control study* - For matched studies, give matching criteria and the number of controls per case	Methods - Study intervention, paragraph 2		
Variables	7	Clearly define all outcomes, exposures, predictors, potential confounders, and effect modifiers. Give diagnostic criteria, if applicable.	Methods – Outcomes and Data sources, paragraph 1, 3	Explain your strategy for improvement and discuss how you implemented your study.	Methods – Study Intervention, paragraph 1
Data sources/ measurement	8	For each variable of interest, give sources of data and			

		details of methods of assessment (measurement). Describe comparability of assessment methods if there is more than one group	Methods – Outcomes and Data sources, paragraph 1, 2, 3		
Bias	9	Describe any efforts to address potential sources of bias	Not described		
Study size	10	Explain how the study size was arrived at	Methods – Statistical Methods, paragraph 1		
Quantitative variables	11	Explain how quantitative variables were handled in the analyses. If applicable, describe which groupings were chosen, and why	Methods – Outcomes and data sources, paragraph 3, - statistical methods, paragraph 1		
Statistical methods	12	(a) Describe all statistical methods, including those used to control for confounding<br>(b) Describe any methods used to examine subgroups and interactions<br>(c) Explain how missing data were addressed<br>(d) *Cohort study* - If applicable, explain how loss to follow-up was addressed<br>*Case-control study* - If applicable, explain how matching of cases and controls was addressed<br>*Cross-sectional study* - If applicable, describe analytical methods taking account of sampling strategy<br>(e) Describe any sensitivity analyses	Methods – Statistical Methods, paragraph 1		
**Results**					

Participants	13	(a) Report the numbers of individuals at each stage of the study (*e.g.*, numbers potentially eligible, examined for eligibility, confirmed eligible, included in the study, completing follow-up, and analyzed)<br>(b) Give reasons for non-participation at each stage.<br>(c) Consider use of a flow diagram	Results – paragraph 1 – Clinically non-indicated (completed) orders, paragraph 1<br>("participants" are "orders")		
Descriptive data	14	(a) Give characteristics of study participants (*e.g.*, demographic, clinical, social) and information on exposures and potential confounders<br>(b) Indicate the number of participants with missing data for each variable of interest<br>(c) *Cohort study* - summarize follow-up time (*e.g.*, average, and total amount)	N/A, there are no participants in this study, the participants are the orders		
Outcome data	15	*Cohort study* - Report numbers of outcome events or summary measures over time<br>*Case-control study* - Report numbers in each exposure category, or summary measures of exposure<br>*Cross-sectional study* - Report numbers of outcome events or summary measures	Results – Clinically non-indicated (completed) orders, paragraph 1, - *C. difficile* HAI, paragraph 1, 2 - *C. difficile* antimicrobial days of therapy, paragraph 1		
Main results	16	(a) Give unadjusted estimates and, if applicable, confounder-adjusted estimates and their precision (e.g., 95% confidence interval). Make	Results – Clinically non-indicated (completed) orders, paragraph 1, - *C. difficile* HAI, paragraph 1	Provide a summary of what your results showed. Comment on whether there were any unintended consequences such as	Results, all paragraphs

		clear which confounders were adjusted for and why they were included<br>(b) Report category boundaries when continuous variables were categorized<br>(c) If relevant, consider translating estimates of relative risk into absolute risk for a meaningful time period		unexpected benefits, problems, failures, or costs associated with the intervention(s).	
Other analyses	17	Report other analyses done—e.g., analyses of subgroups and interactions, and sensitivity analyses	Results – *C. difficile* HAI, paragraph 2		
**Discussion**					
Key results	18	Summarize key results with reference to study objectives	Discussion – paragraph 1	Comment on the strengths of the project. Describe any problems you faced and how you navigated these.	Discussion – paragraph 2, 6
Limitations	19	Discuss limitations of the study, taking into account sources of potential bias or imprecision. Discuss both direction and magnitude of any potential bias	Discussion – paragraph 7	Reflect on your project's limitations.	Discussion – paragraph 7
Interpretation	20	Give a cautious overall interpretation of results considering objectives, limitations, multiplicity of analyses, results from similar studies, and other relevant evidence	Discussion – paragraph 2, 3, 4, 5	Describe whether chance, bias, or confounding have affected your results and whether there was any imprecision in the design or analysis of the project. Are more data points required?	Discussion – paragraph 6, 7
Generalizability	21	Discuss the generalizability (external validity) of the study results	Discussion – paragraphs 3, 6	Comment on the limits of generalizability.	Discussion – paragraphs 3, 6
