# Supplementary material for: Diagnostic stewardship for Clostridioides difficile testing in an acute care hospital: A quality improvement intervention
Source: Antimicrob Steward Healthc Epidemiol. 2023 Apr 5;3(1):e67. doi: 10.1017/ash.2023.141 (PMC10127245; doi:10.1017/ash.2023.141)
Supplement: Supplementary file 1 [file ashsup.zip › S2732494X23001419sup002.pdf]

SUPPLEMENTAL MATERIAL

Diagnostic stewardship for *Clostridioides difficile* testing in an acute care hospital: A quality improvement intervention

Table of Contents

Quality Improvement Observations of our Study 2

Table S1: Frequency of completed *Clostridioides difficile* orders that do not meet clinical criteria for testing during the intervention, weekly by hospital. 3

Table S2. *Clostridioides difficile* hospital-acquired infections in the pre-intervention and intervention periods, by hospital. 5

Quality Improvement Observations of our Study

The quality improvement team leading this work made the following observations about implementation of a diagnostic stewardship intervention to reduce clinically non-indicated *C. difficile* testing.

Implementation: Facility preparation for the intervention

- Infection prevention
  - Project team brainstormed intervention process with input from physician prescribers of various disciplines at the facility, as well as the Antimicrobial Stewardship service
  - Project team presented the process to the infection prevention team during scheduled meetings for process feedback, orientation, and ongoing coaching
- Project team meetings with physician leadership to obtain buy-in
- Laboratory coordination
  - Planning specimen handling: The lab would hold samples for processing until they received a list via email from infection prevention at 3pm each day. Samples that were approved would be processed the next morning. Samples that were not approved were discarded.
  - Laboratorian training in the process
  - Periodic check-in to review and correct deviations from the process
- Educated providers on process
  - Email notification from physician leadership to all physician and advanced practice provider prescribers
  - Intervention presented at hospital infection control committee meeting
- Educated unit-based nurses on process
  - Information on intervention included in weekly updates sent out by nursing leadership

Cost: Estimates of infection preventionist time

- Infection prevention performed the reviews between one and three pm each day, seven days a week. One infection preventionist would perform the reviews each week and weeks would be rotated through twelve infection preventionists.
- The time it took to review all orders from the 24 hours prior varied, but on average approximately two hours each day. This resulted in 14 hours of IP time used each week on *C. difficile* order reviews. Across our two hospitals, there was an average of 85 orders reviewed each week.

Qualitative responses to the intervention

- Providers had improved recognition of ordering criteria and modification to patient care (for example, stopping laxatives and observing prior to testing).
- Provider concerns were addressed by infection preventionists and if necessary, the director of infection prevention or physician leadership. Examples of concerns included: risk of a “missed diagnosis”, a delay in diagnosis, or vulnerability of patients to complications of *C. difficile* (e.g., immunocompromised).

Table S1: Frequency of completed *Clostridioides difficile* orders that do not meet clinical criteria for testing during the intervention, weekly by hospital.

Week Number	Date Range	Hospital A Completed Orders			Hospital B Completed Orders			Total
		Orders not meeting criteria	Total Orders	Percent of orders not meeting criteria	Orders not meeting criteria	Total Orders	Percent of orders not meeting criteria	
1	1/10/22 - 1/16/22	1	26	3.8%	1	13	7.7%	39
2	1/17/22 - 1/23/22	3	26	11.5%	4	22	18.2%	48
3	1/24/22 - 1/30/22	2	32	6.3%	1	24	4.2%	56
4	1/31/22 - 2/6/22	2	21	9.5%	1	14	7.1%	35
5	2/7/22 - 2/13/22	0	27	0.0%	0	26	0.0%	53
6	2/14/22 - 2/20/22	5	24	20.8%	3	30	10.0%	54
7	2/21/22 - 2/27/22	3	39	7.7%	2	23	8.7%	62
8	2/28/22 - 3/6/22	10	26	38.5%	7	20	35.0%	46
9	3/7/22 - 3/13/22	1	23	4.3%	3	22	13.6%	45
10	3/14/22 - 3/20/22	4	33	12.1%	2	29	6.9%	62
11	3/21/22 - 3/27/22	2	30	6.7%	0	18	0.0%	48
12	3/28/22 - 4/3/22	5	20	25.0%	1	16	6.3%	36
13	4/4/22 - 4/10/22	6	22	27.3%	1	20	5.0%	42
14	4/11/22 - 4/17/22	3	33	9.1%	2	20	10.0%	53
15	4/18/22 - 4/24/22	4	38	10.5%	1	18	5.6%	56
16	4/25/22 - 5/1/22	2	27	7.4%	0	27	0.0%	54
17	5/2/22 - 5/8/22	1	42	2.4%	0	21	0.0%	63
18	5/9/22 - 5/15/22	0	25	0.0%	0	13	0.0%	38
19	5/16/22 - 5/22/22	3	32	9.4%	2	15	13.3%	47
20	5/23/22 - 5/29/22	3	37	8.1%	7	27	25.9%	64
21	5/30/22 - 6/5/22	2	25	8.0%	3	26	11.5%	51
22	6/6/22 - 6/12/22	1	28	3.6%	3	24	12.5%	52
23	6/13/22 - 6/19/22	2	28	7.1%	2	26	7.7%	54
24	6/20/22 - 6/26/22	0	24	0.0%	3	19	15.8%	43
25	6/27/22 - 7/3/22	4	29	13.8%	0	18	0.0%	47
26	7/4/22 - 7/10/22	3	33	9.1%	1	22	4.5%	55
27	7/11/22 - 7/17/22	0	26	0.0%	3	25	12.0%	51

28	7/18/22 - 7/24/22	1	25	4.0%	2	25	8.0%	50
29	7/25/22 - 7/31/22	0	23	0.0%	0	26	0.0%	49
30	8/1/22 - 8/7/22	0	25	0.0%	0	25	0.0%	50
31	8/8/22 - 8/14/22	3	29	10.3%	1	22	4.5%	51
32	8/15/22 - 8/21/22	1	25	4.0%	1	28	3.6%	53
33	8/22/22 - 8/28/22	1	27	3.7%	1	23	4.3%	50
34	8/29/22 - 9/4/22	0	17	0.0%	1	22	4.5%	39
35	9/5/22 - 9/11/22	0	22	0.0%	2	22	9.1%	44
36	9/12/22 - 9/18/22	3	28	10.7%	2	20	10.0%	48
37	9/19/22 - 9/25/22	1	23	4.3%	1	20	5.0%	43
38	9/26/22 - 10/2/22	0	34	0.0%	0	15	0.0%	49
39	10/3/22 - 10/9/22	0	25	0.0%	0	22	0.0%	47
40	10/10/22 - 10/14/22	0	18	0.0%	0	13	0.0%	31
Total		**82**	**1097**	7.5%	**64**	**861**	7.4%	**1958**

** 21 completed orders that were processed without infection prevention approval are excluded in this table*

Table S2. ***Clostridioides difficile*** **hospital-acquired infections in the pre-intervention and intervention periods, by hospital.**

	Pre-Intervention Period											Intervention Period									
	Mar	Apr	May	Jun	Jul	Aug	Sep	Oct	Nov	Dec	Jan 1-9	Jan 10-31	Feb	Mar	Apr	May	Jun	Jul	Aug	Sep	Oct 1-14
**Hospital A**	17	18	17	10	17	14	12	12	15	11	8	6	15	13	14	20	9	15	12	16	6
**Hospital B**	11	12	16	11	7	17	8	11	17	15	3	9	6	9	10	10	8	12	8	14	3
**Total**	28	30	33	21	24	31	20	23	32	26	11	15	21	22	24	30	17	27	20	30	9

**Two HAIs were reported during the intervention period from orders placed at an outside facility.*
